# A revision of *Megalocraerus* Lewis, 1902 (Coleoptera, Histeridae: Exosternini)

**DOI:** 10.3897/zookeys.557.7087

**Published:** 2016-01-28

**Authors:** Michael S. Caterino, Alexey K. Tishechkin

**Affiliations:** 1 Department of Agricultural & Environmental Sciences, Clemson University, Clemson, SC 29634-0310 USA; 2 USDA Systematic Entomology Laboratory, c/o Smithsonian Institution, National Museum of Natural History, Washington, DC 20013-7012

**Keywords:** Histeridae, Histerinae, Exosternini, subcortical predator, Neotropical region

## Abstract

The formely monotypic Neotropical genus *Megalocraerus* Lewis is revised to include five species, known from southeastern Brazil to Costa Rica: *Megalocraerus rubricatus* Lewis, *Megalocraerus mandibularis* **sp. n.**, *Megalocraerus chico* **sp. n.**, *Megalocraerus madrededios* **sp. n.**, and *Megalocraerus tiputini* **sp. n.** We describe the species, map their distributions, and provide a key for their identification. Their subcylindrical body form and emarginate mesosternum have previously hindered placement to tribe, although their curent assignment to Exosternini now appears well supported by morphological evidence. Nothing is known of the natural history of the species.

## Introduction

*Megalocraerus* Lewis is a rarely-collected, hitherto monotypic genus of Histerinae, occurring only in the Neotropical realm, of historically uncertain placement. When Lewis (1902) established the genus, uncertainty about its relationships was already evident in his comparison to various genera: “The genus established here is represented by a species having the superfices of *Pachycraerus* [Marseul], but the mesosternum is not produced anteriorly and the tarsal grooves in the anterior tibiae are straight. The antennal fossettes are similar to those of *Exosternus*” (Lewis 1902). Lewis’s ‘superfices of *Pachycraerus*’ most likely referred to the elongate, subcylindrical body shape shared by these two genera. When Bickhardt (1917) established tribes in Histerinae, *Megalocraerus* was placed in Histerini, while the other two genera were placed in Exosternini, defined by the projecting mesoventrite *Megalocraerus* had been noted to lack. The genus has received little further attention, and has remained in Histerini since, through several recent catalogs (Mazur 1984, 1997, 2011).

There have been few attempts to refine tribes in Histerinae, and none have attempted to distinguish symplesiomorphies from synapomorphies. Diagnoses based on single characters, such as presence or absence of a projecting mesoventrite, exemplify this ambiguity. According to recent treatments, Histerini includes those Histerinae with the anterior margin of the mesoventrite emarginate or straight (Bickhardt 1917), the protarsal grooves straight (Bickhardt 1917), and two complete antennal annuli present (Mazur 1990). While *Megalocraerus* satisfies the first two of these, no more than a single complete antennal annulus can be seen. However, the elongate subcylindrical shape of *Megalocraerus* is otherwise unknown in Histerini. *Megalocraerus* is excluded from either Platysomatini or Omalodini by its lack of distinctly ‘V’-shaped antennal annuli (Mazur 1990). Mazur’s (1990) definition of Exosternini relied only on reduced antennal annuli and a ‘simple, tubular’ penis, implicitly discarding the shape of the anterior mesoventral margin as significant. Despite the fact that this loose definition of Exosternini could have included *Megalocraerus*, it has never been reassigned.

Recent attempts to resolve phylogenetic relationships in Exosternini and Histerinae (Caterino and Tishechkin 2015, Leivas et al. 2015) have mostly served to show that none of the tribes of Histerinae are monophyletic as currently constituted (perhaps true even of the subfamily). However, *Megalocraerus* is closely related to most other Neotropical Exosternini, with particularly close relationships to *Hypobletus* Schmidt. Among their putative synapomorphies, some of the most reliable would be elongate body form, emarginate mesoventrite, emarginate epistoma, antennal club with reduced annuli, and apices of elytra with increasingly distinct punctures (reconstructed as a parallelism with *Baconia*, in which this condition is also distinct). Noteably, the Afrotropical genera suggested by Lewis (1902) to show some commonalities, *Pachycraerus* and *Exosternus*, are far removed (all results from Caterino and Tishechkin 2015).

In addition to hereby formally reassigning *Megalocraerus* to Exosternini (at least pending further work on tribal definitions of Histerinae), we herein describe several newly discovered species in the genus, which serve to broaden its scope and morphological diversity considerably.

## Materials and methods

The morphological terminology used follows Caterino and Tishechkin (2014), based on Wenzel and Dybas (1941), Helava et al. (1985), Ôhara (1994) and Lawrence et al. (2011). Total body length is measured from the anterior margin of the pronotum to the posterior margin of the elytra (to exclude preservation variability in head and pygidial extension); width is measured at the widest point, generally near the elytral humeri. Conventional imaging was done using a Visionary Digital’s ‘Passport’ portable imaging system, which incorporates a Canon 7D with MP-E 65mm 1–5× macro zoom lens. Images were stacked using Helicon Focus software (www.heliconsoft.com). SEM imaging was done on a Zeiss EVO 40 scope, and the specimen was sputter coated with gold.

**Collections are abbreviated as follows**:

BMNH
Natural History Museum, London, UK 


CHND
 The Nicolas Dégallier collection, Paris, France 


FMNH
 The Field Museum, Chicago, USA 


FSCA
 Florida State Collection of Arthropods, Gainesville, USA 


INBIO
Instituto Nacional de Biodiversidad, San Jose, Costa Rica 


SEMC
 Snow Entomology Museum, University of Kansas, Lawrence, USA 


UFPR
Universidade Federal do Paraná, Curitiba, Brazil 


## Taxonomy

### Megalocraerus

Taxon classificationAnimaliaColeopteraHisteridae

Lewis, 1902

Megalocraerus Lewis, 1902: 231

#### Type species.

*Megalocraerus rubricatus* Lewis, by monotypy.

#### Diagnosis.

The genus is readily separated from other Histerinae in the Neotropics, where few other large cylindrical species have been described. In addition to this general body shape, the depressed epistoma and reduced, subtriangular labrum will distinguish any member of the genus easily.

#### Description.

***Size range***: Length 2.8–4.1 mm; width 2.0–2.6 mm; ***Body***: elongate, parallel-sided, subcylindrical, slightly depressed; body castaneous to piceous, rarely bicolored with the elytral bases rufescent. ***Head***: Frons broad, prominent, slightly bulging in front of eyes, becoming depressed and narrowing toward concave epistoma; frontal disc coarsely punctate; frontal stria deeply impressed along inner edges of eyes, angled anteromediad at front, interrupted across width of epistoma, free ends may be bent mediad or dorsad; supraorbital stria largely obsolete, fragments may be present at middle; labrum reduced, short and subtriangular, apex rounded to subangulate; mandibles rather short, more or less evenly curved to subacute apices, usually with prominent tooth on each incisor edge, lacking inner submarginal ridge; antennal scape moderately expanded to apex, with carina along outer posterior edge, funicle gradually expanded, antennomere 8 broadly cupuliform; antennal club rounded, not elongate, apex rounded to subtruncate, with apical and subapical annuli crowded into apical third, subapical annulus weakly expanded at middle of dorsal and ventral surfaces, basal annulus absent; gena narrowly depressed posterad cardo; gular sutures visible but not impressed; submentum short, with apical row of ~15 setae; mentum narrowing anterad, apex emarginate, with sparse setae; labial and maxillary palpi basically fusiform, narrowed apically. ***Pronotum***: Pronotal sides subparallel, weakly rounded, narrowed anteriorly, apical emargination broad; vaguely but indistinctly depressed in prescutellar region; anterior gland openings very fine, present on sides of anterior emargination; marginal stria complete along sides, variably fragmented to obsolete along anterior margin; marginal pronotal stria very fine, continuous around lateral and anterior margins. ***Elytra***: two (rarely three) complete epipleural striae present, innermost continued variably mediad along part of anterior elytral margin; inner and outer subhumeral striae at most weakly indicated, generally absent; striae 1-4 complete, 5^th^ stria complete or basally abbreviated; sutural stria complete, connected to base of 5^th^ or rarely 4^th^ stria; elytral disc with coarser punctation toward apex, ground punctation usually very fine and sparse but may be coarser and more conspicuous; elytral striae tending to become prolonged mediad along the basal elytral margin, sometimes forming a distinct stria. ***Prosternum***: Prosternal lobe broad, about two-thirds length of keel; with marginal stria variably obsolete at sides; prosternal keel weakly produced posteriorly, with two complete carinal striae free or joined at apices; lateral striae of keel widely divergent anterad along presternal suture. ***Mesoventrite***: Anterior mesoventral margin broadly, not too deeply emarginate, with complete marginal stria, often with secondary fragments in anterolateral corners; mesometaventral stria absent. ***Metaventrite***: Postmesocoxal stria short or indisinct; inner lateral metaventral stria variably abbreviated apically, not reaching metacoxa; disc with only fine ground punctation. ***Abdomen***: Abdominal ventrite 1 usually with one more or less complete lateral stria, anterior margin lacking stria; propygidium short, 4–5× broader than long, disc lacking obvious gland openings; pygidium with apical margin broadly rounded, lacking marginal stria. ***Legs***: Protrochanter lacking setae; meso- and metatrochanters each with two very short setae (often abraded or lost); protibia acutely multidentate, with 5–6 lateral marginal spines and 2–3 fine apical marginal spines; two protibial spurs present, short; tarsal groove on anterior surface only well developed in apical fourth; protarsi not sexually dimorphic, with single pair of apical setae on most tarsomeres; mesotibial margin distinctly multidentate, with 3–6 marginal denticles; metatibial margin not toothed, but with several fine marginal spines, tarsal claws simple; apical tarsomere on all tarsi elongate, curved; ventral tarsal setae variable, simple to flattened and sublaminate. ***Male***: accessory sclerites absent; 8^th^ tergite subparallel-sided to narrowed apically, basal apodemes of 8^th^ tergite with thin, acute ventral extensions curving distad, convergent medially, separated from body of tergite by deep lateral emarginations parallel to basal margin; ventrolateral lobes rounded to subacute, variably separated along ventral midline; 8^th^ sternite divided along midline with inner edges parallel, more or less approximate, outer upper edges produced apically into narrow, convergent processes or broad lobes, apices with thin velar membrane between, lacking distinct setae; 9^th^ tergite with apices narrow, roundly convergent, lacking setae, ventrolateral apodemes weak to distinctly dentate; spiculum gastrale (S9) generally wide-stemmed, relatively weakly sclerotized along midline, apices divergent but not strongly produced or hooked; 10^th^ tergite entire or variously partially divided, usually evenly sclerotized but sometimes appearing more strongly sclerotized toward apex; basal piece about one-fourth tegmen length, with apicoventral apodemes produced, more or less convergent beneath base of tegmen; tegmen rather narrow, moderately to strongly dorsoventrally flattened, usually lacking medioventral process, apices divided, sometimes distinctly separated at tips; median lobe half or greater length of tegmen, with proximal arms bent near midpoint. ***Female***: 8^th^ tergite broad, relatively flat, deeply emarginate apically; 8^th^ sternite undivided, with strongly sclerotized, straight basal apodemes; valviferae weakly expanded proximally; coxite simple, only weakly differentiated into upper and lower surfaces, lacking apical teeth, apex narrowed, subtruncate, with prominent, setose gonostyle; bursa copulatrix membraneous; spermatheca forming a short, bulbous sac, inserted beneath apex of bursa, bearing single thin spermathecal gland near its base.

#### Distribution.

This genus is known from Central and South America, from Costa Rica in the north to Rio de Janeiro state (Brazil) in the south. However, there are many gaps, with records only from Costa Rica, French Guiana, Ecuador, Peru, and Brazil, though it almost certainly occurs in the intervening areas.

#### Natural history.

No specimen labels reveal any details of the natural history of the species of *Megalocraerus*, with all or nearly all specimens collected using flight interception (‘window’) traps. Given the subcylindrical, elongate morphology, an under-bark habit may be suggested, but there is no direct evidence for any such association.

### Key to species

**Table:** 

1	Elytra with rufescent maculations; prosternal carinal striae subparallel and relatively narrowly separated	***Megalocraerus rubricatus* Lewis**
–	Body unicolorous, black to castaneous; prosternal carinal striae divergent at least posteriorly	**2**
2	Ground punctation of elytra conspicuous, with coarse secondary punctures gradually becoming denser in apical half	**4**
–	Ground punctation of elytra fine and largely inconspicuous; coarser apical punctures rather discretely limited to about apical one-fifth	**3**
3	Male with dorsobasal mandibular processes; ventral setae of tarsomeres simple; Guianas	***Megalocraerus mandibularis* sp. n.**
–	Male mandible unmodified; ventral setae of all tarsomeres flattened, sublaminate; Central America	***Megalocraerus chico* sp. n.** *
4	Body broader, shorter and flatter, vaguely rufescent; 4^th^ dorsal stria united with sutural stria at base, 5^th^ stria slightly abbreviated at base; known from Peru	***Megalocraerus madrededios* sp. n.**
–	Body more elongate and more convex, piceous; 5^th^ dorsal stria united with sutural stria at base; known from Ecuador	***Megalocraerus tiputini* sp. n.**

*A possibly distinct form known from French Guiana keys out here. However, we have no males to properly assess its distinctness and do not describe it here.

### Megalocraerus rubricatus

Taxon classificationAnimaliaColeopteraHisteridae

Lewis, 1902

**Figure 1.**
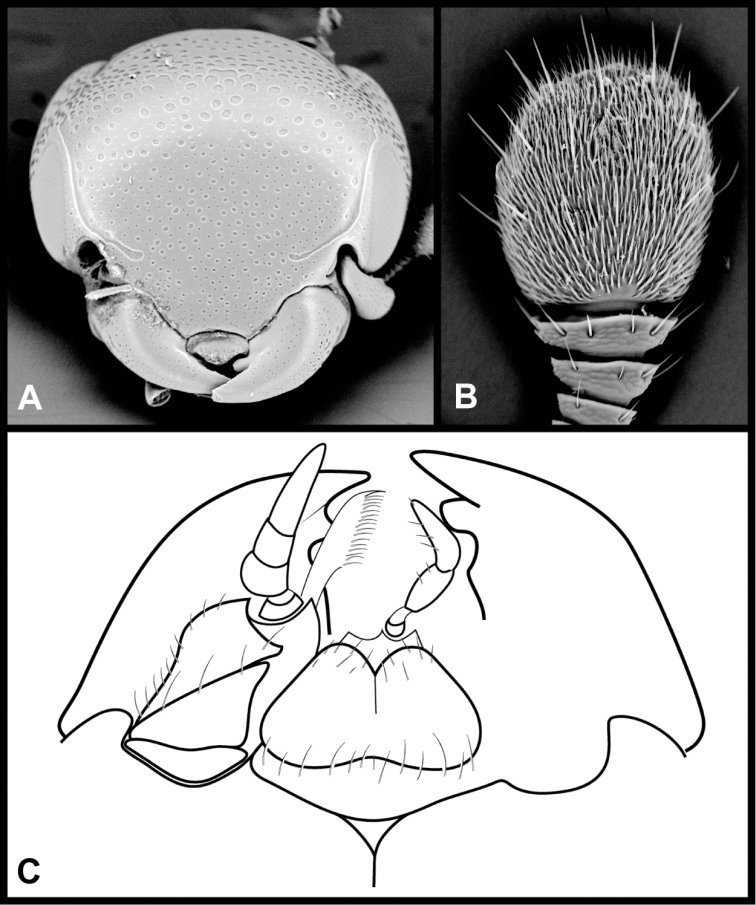
Generic characters of *Megalocraerus*. **A** Frons **B** Antenna **C** Mouthparts, ventral view (one maxilla and labial palpus omitted for clarity).

**Figure 2.**
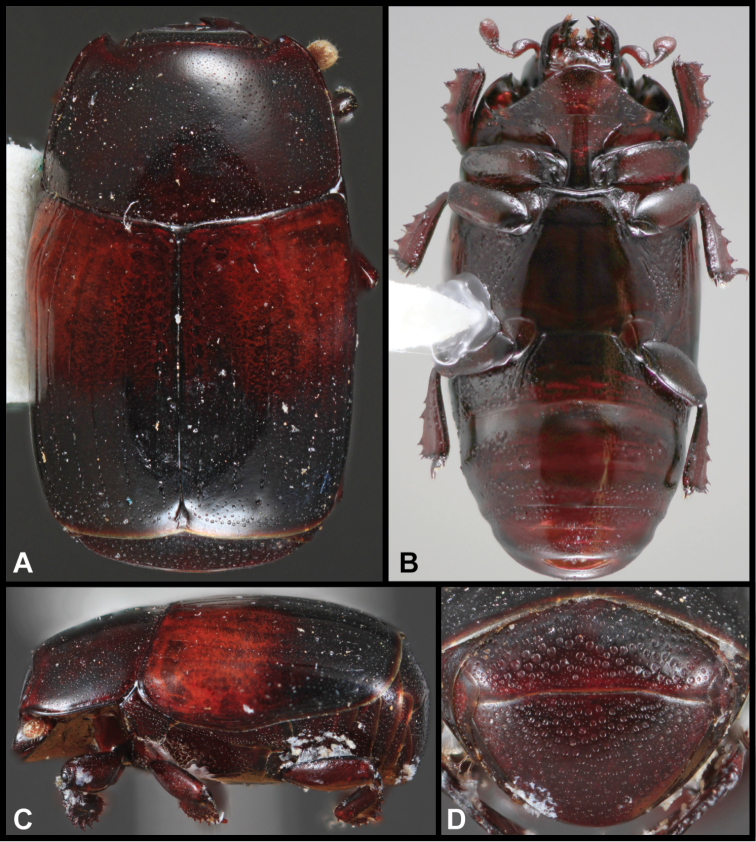
Lectotype of *Megalocraerus rubricatus*. **A** Dorsal **B** Ventral **C** Lateral **D** Pygidial habitus.

**Figure 3.**
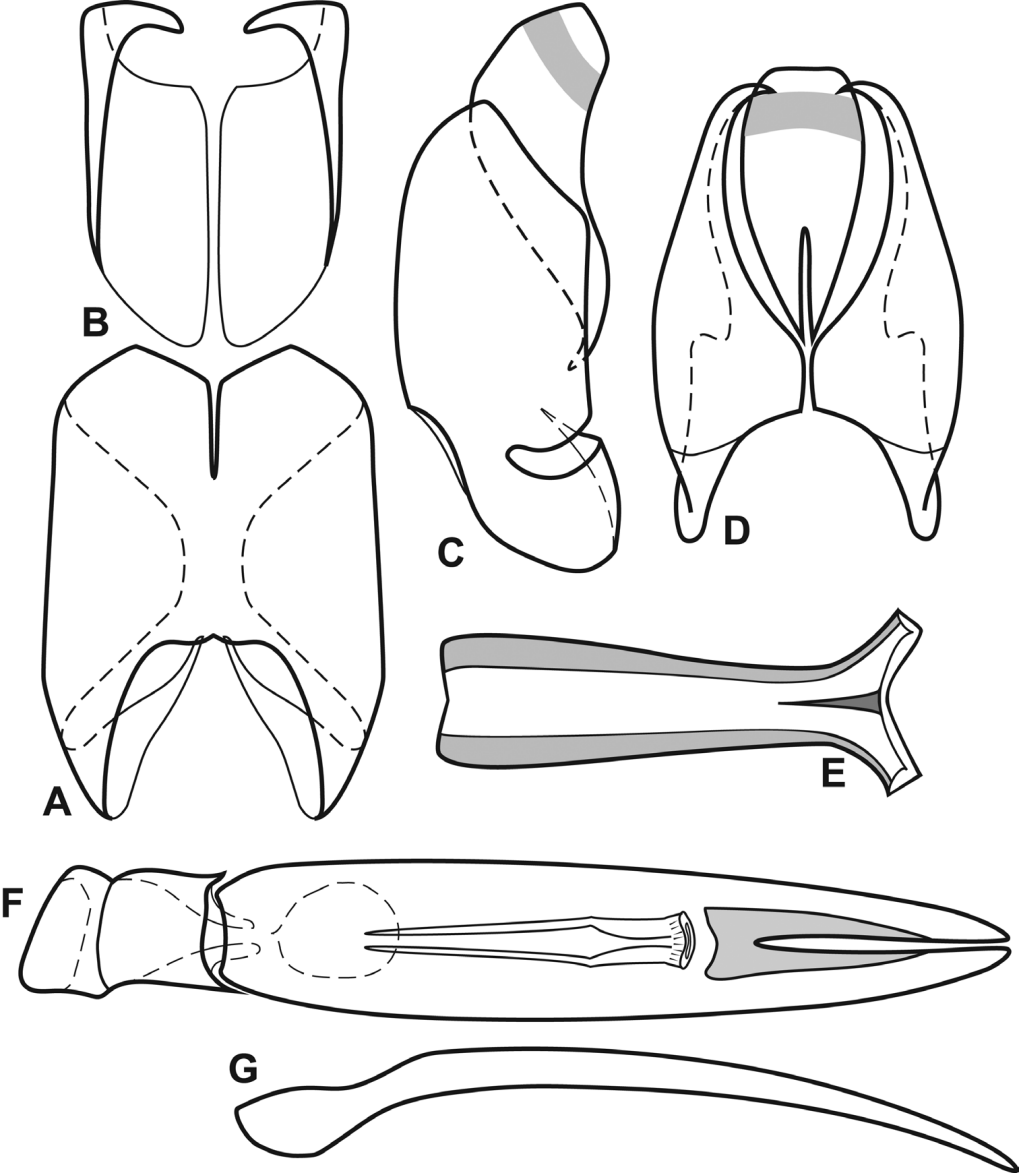
Male genitalia, *Megalocraerus rubricatus*. **A** 8^th^ tergite, dorsal view **B** 8^th^ sternite, dorsal view **C** 8^th^ tergite and sternite, lateral view, *in situ* **D** 9^th^ and 10^th^ tergites, dorsal view **E** 9^th^ sternite, dorsal view **F** Aedeagus, dorsal view **G** Tegmen, lateral view.

**Figure 4.**
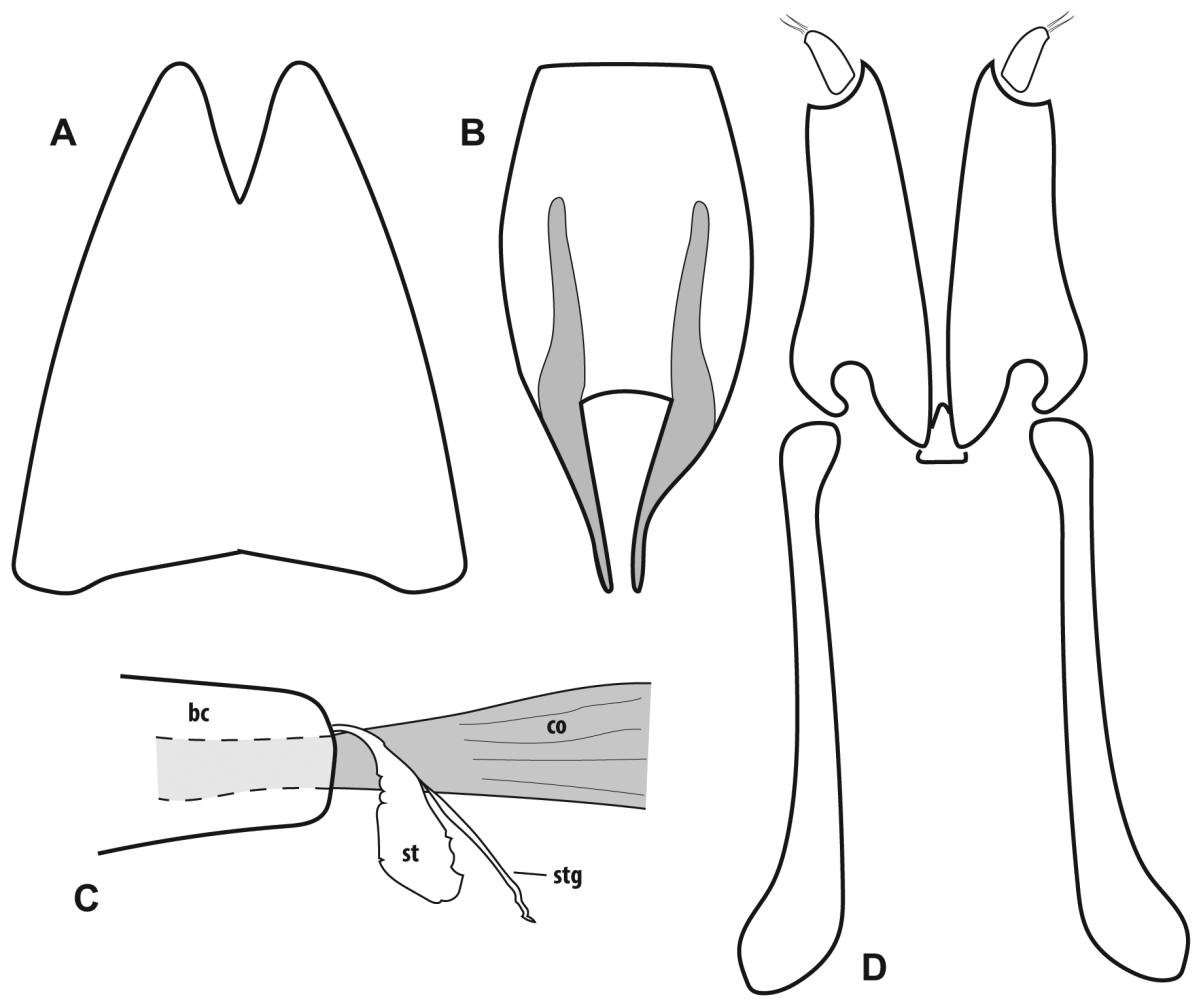
Female genitalia, *Megalocraerus rubricatus*. **A** 8^th^ tergite, dorsal view **B** 8^th^ sternite, ventral view **C** Bursa copulatrix (bc), common oviduct (co), spermatheca (st) and attached spermathecal gland (stg) **D** Ovipositor.

Figs 1 , 2 , 3 , 4 , 8

Megalocraerus rubricatus Lewis, 1902: 231

#### Type material.

**Lectotype**, here designated (BMNH): “Bresil”/”Jatahy, Prov. Goyas”. Although Lewis slightly differentiated the primary type locality in Goyas, his mention of a second specimen from ‘the Amazon Region’ leaves some room for ambiguity, which we address through this lectotype designation; cotype only “Bresil”.

#### Other material.

**Brazil** (country record only; ‘Amazon Region’ as published; Lewis, 1902; BMNH). **French Guiana**: 1: Rés. des Nouragues, Régina, 4°2.27'N, 52°40.35'W, 10.x.2009, FIT, SEAG (CHND); 4: same locality and collectors, 3.xi.2009, (CHND); 1: Belvèdére de Saül, 3°1'22"N, 53°12'34"W, FIT, 17.ix.2010, SEAG (CHND).

#### Diagnosis.

The type species is the most easily recognizeable in the genus, being at once the largest and most elongate, while also unique in its red basal elytral maculae. The basal sutural arch does not meet any of the other dorsal striae, whereas it is continuous with either the 4^th^ or 5^th^ dorsal stria in all other species.

#### Description.

***Size***: Length 3.5–4.1 mm; width 2.3–2.6 mm; ***Body***: elongate, parallel-sided, moderately depressed; castaneous with distinct red maculations on basal two-thirds of elytra. ***Head***: Frons finely and doubly punctate, with medium punctures separated by about their diameters against fine ground punctation, decreasing in size but slightly increasing in density anteromediad; frontal stria present along inner margins of eyes, broadly interrupted across epistoma; supraorbital stria absent; epistoma depressed, narrowing anterad; labrum impunctate, but with fine microsculpture; mandibles each with inner marginal tooth. ***Pronotum***: Pronotal sides weakly rounded, slightly narrowed anterad, marginal stria complete, lateral stria absent, fragments of anterior stria usually present; pronotal disk with small secondary punctures sparse basomedially, increasing in density toward front and sides, with fine ground punctation more or less uniform; larger punctures present along posterior margin. ***Elytra***: Two complete epipleural stria present; subhumeral striae absent; dorsal striae 1–4 more or less complete, 5^th^ stria obsolete near base, not meeting basal arch of complete sutural stria; bases of dorsal striae extending mediad along basal elytral margin, but only very rarely meeting base of next stria; ground punctation of elytral disk fine, small secondary punctures present in apical fourth. ***Prosternum***: Prosternal lobe evenly rounded, complete to sides, with marginal stria usually obsolete at sides; prosternal keel with two complete carinal striae. ***Mesoventrite***: Anterior mesoventral margin evenly emarginate, with complete, fine marginal stria; mesometaventral stria absent; disk with only fine ground punctation. ***Metaventrite***: Metaventral disk finely punctate at middle, more coarsely so laterad lateral metaventral stria; postmesocoxal strial obscured by punctation. ***Abdomen***: Abdominal ventrites rather coarsely but shallowly punctate throughout widths; propygidium transverse, about three times as wide as long, coarsely punctate, with punctures separated by slightly less than their diameters; pygidium similarly coarsely punctate at base, more finely and sparsely apicad. ***Male***: 8^th^ tergite with deep, rather narrow basal emargination, ventrobasal processes nearly meeting, dorsally with fine median emargination; halves of 8^th^ sternite approximate along much of midline, apicomedial processes rather narrow, subacute; 9^th^ tergite with acute, incurved apices; spiculum gastrale (S9) broad throughout, slightly narrowed at neck, apex shallowly emarginate; 10^th^ tergite with median basal emargination; aedeagus flattened throughout, sides weakly rounded, apices slightly separated; median lobe slightly more than one-half tegmen length.

#### Remarks.

While previously the only exemplar of the genus, *Megalocraerus rubricatus* is atypical of the group in a number of ways, being significantly larger, flatter, and with distinctive coloration. It is easily recognized in the group.

### Megalocraerus mandibularis sp. n.

Taxon classificationAnimaliaColeopteraHisteridae

http://zoobank.org/BA792625-172B-4467-ABD4-E693BAB30CD9

**Figure 5.**
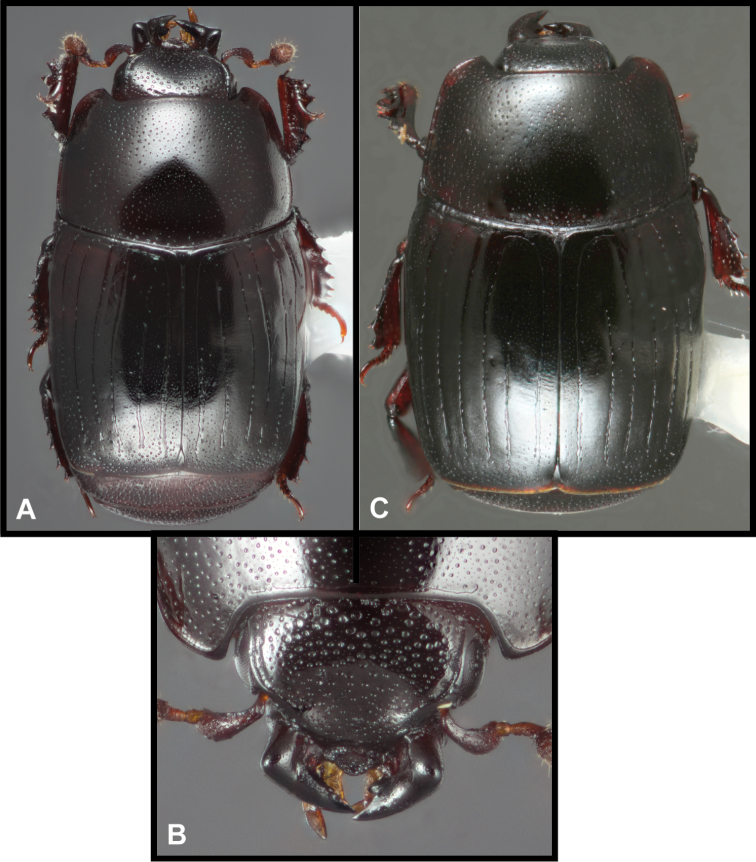
**A** Dorsal habitus *Megalocraerus mandibularis* **B** Mandibles male *Megalocraerus mandibularis* **C** Dorsal habitus *Megalocraerus chico*.

**Figure 6.**
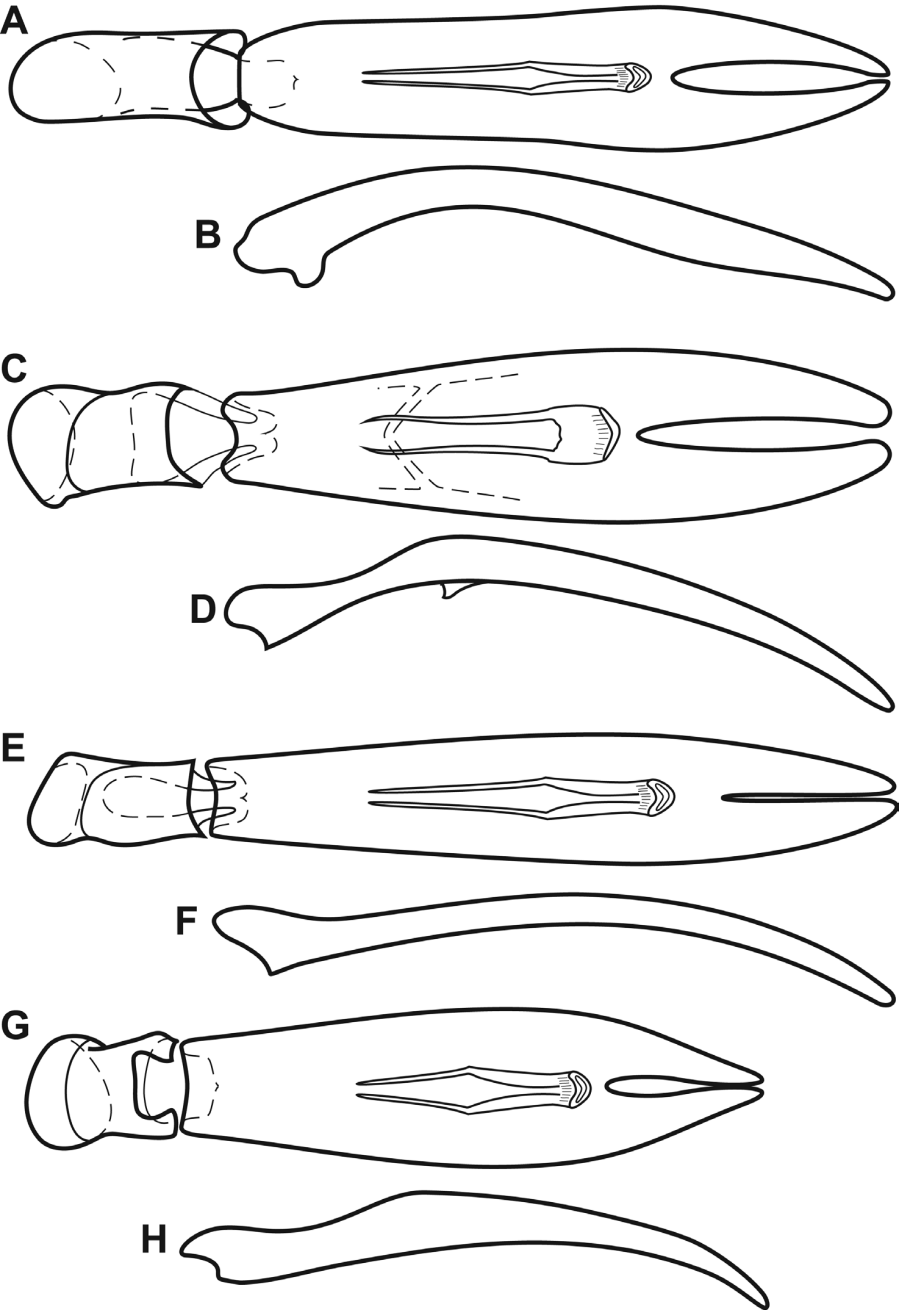
Aedeagi of *Megalocraerus* spp. **A** *Megalocraerus mandibularis*, dorsal view **B** *Megalocraerus mandibularis*, lateral view **C** *Megalocraerus chico*, dorsal view **D** *Megalocraerus chico*, lateral view **E** *Megalocraerus madrededios*, dorsal view **F** *Megalocraerus madrededios*, lateral view **G** unnamed *Megalocraerus* sp. from Rio de Janeiro, dorsal view **H** unnamed *Megalocraerus* sp. from Rio de Janeiro, lateral view.

Figs 5A–B , 6A–B , 8

#### Type material.

**Holotype male**: “FRENCH GUIANA, KAW, xii 2014, leg: J.L.GUIGLARIS” (FMNH).

#### Diagnosis.

This species is very distinctive, at least in the male, exhibiting unique dorsobasal mandibular processes (Fig. 6). Because only the male is known, it is unclear if these are secondary sexual characters or not, though it seems likely. Otherwise the species is very similar to *Megalocraerus chico* known from southern Central America. The two differ in tarsal setae, with *Megalocraerus mandibularis* having simple ventral setae, while *Megalocraerus chico* has sublaminate setae. In addition, *Megalocraerus mandibularis* has rather more numerously and strongly dentrate tibiae, than any other species, with the mesotibia in particular exhibiting 6 distinct spinose teeth. Other species generally show fewer and less deeply divided tibial teeth on all legs. Finally, the basal elytral stria, uniting the bases of the marginal epipleural through sutural dorsal striae, is more strongly and completely impressed here than in other species, although the extent of variation in this character in other species is difficult to assess with limited material. The male genitalia are highly distinct, showing strong dorsoventral curvature.

#### Description.

***Size***: Length 2.8 mm; width 2.0 mm; ***Body***: broad, subparallel-sided, elongate, weakly depressed; darkly castaneous. ***Head***: Frons finely and doubly punctate, with medium punctures separated by slightly more than their diameters against fine ground punctuation, decreasing in size but slightly increasing in density anteromediad, sparser toward the eyes; frontal stria present along inner margins of eyes, bent inward above antennal insertions, broadly interrupted across epistoma; faint fragments of supraorbital stria present; epistoma depressed, narrowing anterad; labrum minutely punctate; mandibles each with inner marginal tooth and (in male) with bluntly triangular dorsobasal process. ***Pronotum***: Pronotal sides weakly convergent to anterior corners, marginal stria present along sides and front, very briefly interrupted behind eyes; pronotal disk rather finely and sparsely punctate, puncture density diminishing basomedially, with finer ground punctation throughout; basal margin with a few coarser punctures near margin. ***Elytra***: Two complete and an outer partial epipleural striae present; fine fragments of inner subhumeral stria present at middle; dorsal striae 1–4 largely complete, weak or fragmented apically; 5^th^ stria interrupted near basal third, obsolete in basal sixth; sutural stria complete; inner epipleural, 1st-4th and sutural striae connected by distinct basal marginal stria; elytral disks with fine but conspicuous ground punctation throughout, coarser secondary punctures present in apical sixth, with few secondary punctures scattered in apical interstriae, particularly along the elytral suture. ***Prosternum***: Prosternal lobe evenly, somewhat narrow rounded, complete to sides, marginal stria present only at middle for approximately buccal cavity width; prosternal keel with complete carinal striae diverging slightly front and rear, indistinct around basal projection. ***Mesoventrite***: Anterior mesoventral margin evenly emarginate, with complete, marginal stria; mesometaventral stria absent; disk with very fine, sparse ground punctation, with few secondary punctures at sides. ***Metaventrite***: Lateral metaventral stria present, extending toward outer corner of metacoxa, slightly abbreviated apically; postmesocoxal stria indistinct. ***Abdomen***: Abdominal ventrites finely punctate at middle, slightly more coarsely at sides; propygidium strongly transverse, about four times as wide as long, coarsely punctate, with punctures separated by slightly less than their diameters, rather uniformly throughout; pygidium similarly coarsely punctate at base, punctures becoming finer and denser toward apex. ***Male***: 8^th^ tergite with broad basal emargination, ventrobasal processes rather weak, not meeting beneath, dorsally with narrow median emargination; halves of 8^th^ sternite slightly separated along midline, apicomedial processes rather narrow, subacute; 9^th^ tergite with blunt, incurved apices; spiculum gastrale (S9) broad basally and apically, abruptly narrowed near midpoint, apex shallowly emarginate; 10^th^ tergite entire; basal piece about one-third tegmen length, narrowly cylindrical; tegmen flattened throughout, abruptly bent ventrad one-third from base, sides weakly, sinuately divergent from base two-thirds to apex, narrowing to apices; median lobe narrow, slightly more than one-half tegmen length.

#### Remarks.

We mention a ‘French Guiana form’ known only from females (or specimens who lost their genitalia prior to study) in the key and below. There is some slight possibility that it represents the female of this species, based not only on the distribution, but also on the shared character of relatively strongly spinose tibiae. However, the size difference is substantial, with the male *Megalocraerus mandibularis* much smaller than the three known specimens of this other form. Additional material will be necessary to confidently address this question.

This species is named for its unique and prominent mandibular processes.

### Megalocraerus chico sp. n.

Taxon classificationAnimaliaColeopteraHisteridae

http://zoobank.org/A7AB7A75-BEF8-4A4D-A902-7A89389FFD9E

Figs 5C , 6C–D , 8

#### Type material.

**Holotype male**: “Rancho Quemado, Península de Osa, 200 m, Prov. Punt., COSTA RICA. F.Quesada, Dic 1991, L-S 292500, 511000” / “INBIO CRI000331213” (INBIO); **Paratype** female: Turrialba, CATIE [Centro Agronómico Tropical de Investigacion y Enseñanza], 22.vi.-5.vii.1984, H.L.Dozier (FSCA).

#### Diagnosis.

This species is relatively small and dorsoventrally depressed. It is similar overall to *Megalocraerus mandibularis*, but lacks the modified male mandibles of that species. They also differ in tarsal setae, which are flattened and sublaminate in this species, but simple in *Megalocraerus mandibularis*. An additional form that we do not describe from French Guiana keys out with this species, although that entity is significantly larger and has more conspicuous meso- and metaventral punctation. This species and *Megalocraerus madrededios* both have the 4^th^ dorsal stria connected to the sutural arch, but the latter species is more distinctly broad and flattened, as well as lighter in color. Finally, the distinct medioventral process of the aedeagus is unique.

#### Description.

***Size***: Length 2.9–3.0 mm; width 2.0 mm; ***Body***: broad, subparallel-sided, elongate, moderately depressed; piceous to weakly castaneous. ***Head***: Frons finely and doubly punctate, with medium punctures separated by slightly more than their diameters against fine ground punctation, decreasing in size but slightly increasing in density anteromediad; frontal stria present along inner margins of eyes, bent inward above antennal insertions, broadly interrupted across epistoma; supraorbital stria absent; epistoma depressed, narrowing anterad; labrum minutely punctate; mandibles each with inner marginal tooth. ***Pronotum***: Pronotal sides straight, subparallel in basal three-fourths, abruptly narrowed anteriorly, marginal stria complete, lateral stria absent, anterior marginal stria more or less complete; pronotal disk with numerous shallow, elongate secondary punctures at sides and front, diminishing basomedially, with fine ground punctation largely restricted to basal area; basal margin with coarser punctures. ***Elytra***: Two complete epipleural striae present; subhumeral striae absent; dorsal striae 1–4 complete, 1–3 variously continued mediad along basal margin, 4^th^ meeting basal arch of sutural stria, 5^th^ stria obsolete in anterior fourth; elytral disks with conspicuous ground punctation throughout, coarser secondary punctures present in apical sixth. ***Prosternum***: Prosternal lobe evenly, somewhat narrowly rounded, complete to sides, marginal stria obsolete at sides; prosternal keel with two complete carinal striae diverging slightly front and rear, continued around basal projection of keel (may be fragmented). ***Mesoventrite***: Anterior mesosternal margin evenly emarginate, with complete marginal stria; mesometasternal ventral absent; disk with only fine ground punctation. ***Metaventrite***: Metaventral disk impunctate at middle, lateral metaventral stria present, extending toward outer corner of metacoxa, slightly abbreviated apically; postmesocoxal stria very short. ***Abdomen***: Abdominal ventrites finely punctate at middle, slightly more coarsely at sides; propygidium strongly transverse, about four times as wide as long, coarsely punctate, with punctures separated by slightly less than their diameters, rather uniformly throughout; pygidium similarly coarsely punctate at base, punctures becoming finer, but also denser toward apex. ***Male***: 8^th^ tergite with deep but rather broad basal emargination, ventrobasal processes thin, distant beneath, dorsally with fine, deep median emargination, with desclerotized line extending nearly to base; halves of 8^th^ sternite slightly separated along midline, apicomedial processes rather narrow, subacute; 9^th^ tergite with blunt, incurved apices; spiculum gastrale (S9) broad in basal third, abruptly narrowed, thence broadening to head, apex shallowly emarginate; 10^th^ tergite entire; aedeagus flattened throughout, with distinct medioventral process, sides weakly rounded, widest near midpoint, apices slightly separated; median lobe slightly less than one-half tegmen length.

#### Remarks.

The name of this species (Spanish for ‘small’) refers to its relatively small size. In this case it is used as a noun in apposition. This species was included in the phylogenetic analysis of Caterino and Tishechkin (2015) as ‘*Megalocraerus*2’.

### Megalocraerus madrededios sp. n.

Taxon classificationAnimaliaColeopteraHisteridae

http://zoobank.org/DBC355ED-9B57-4039-92C0-76EE483848C8

Figs 6E–F , 7A , 8

#### Type material.

**Holotype male: PERU: Madre de Dios**: Pantiacolla Lodge, Alto Madre de Dios R., 12°39.3'S, 71°13.9'W, 420 m, 14-19.xi.2007, FIT, D. Brzoska (SEMC0874296); Paratype female: same data as type (SEMC0872118); SEMC.

#### Diagnosis.

The body shape of this species is distinct, being broader and less elongate, and slightly more depressed than any of the others. Additionally the 4^th^ stria meeting the basal sutural arch is shared only with *Megalocraerus chico* (above).

#### Description.

***Size***: Length 3.1–3.3 mm; width 2.3–2.4 mm; ***Body***: broad, subparallel-sided, slightly elongate, moderately depressed; castaneous to slightly rufescent; the elytral bases, pronotum, and venter tending to be slightly lighter than the apical elytral two-thirds. ***Head***: Frons finely and doubly punctate, with medium punctures separated by slightly more than their diameters against fine ground punctation, decreasing in size but slightly increasing in density anteromediad; frontal stria present along inner margins of eyes, bent inward above antennal insertions, broadly interrupted across epistoma; supraorbital stria absent; epistoma depressed, narrowing anterad; labrum minutely punctate; mandibles each with inner marginal tooth. ***Pronotum***: Pronotal sides weakly rounded, slightly narrowed anterad, marginal stria complete, lateral stria absent, anterior marginal stria slightly fragmented; pronotal disk with small secondary punctures sparse basomedially, increasing in density toward front and sides, with fine ground punctation more or less uniform. ***Elytra***: Two complete epipleural stria present; subhumeral striae absent; dorsal striae 1–4 complete, 4^th^ meeting basal arch of sutural stria, 5^th^ stria obsolete near base; bases of dorsal striae extending mediad along basal elytral margin, but not meeting base of next stria; elytral disks with conspicuous ground punctation throughout, and increasingly dense secondary punctation toward apices. ***Prosternum***: Prosternal lobe evenly, broadly rounded, complete to sides, with marginal stria obsolete at sides; prosternal keel with two complete carinal striae converging anteriorly. ***Mesoventrite***: Anterior mesoventral margin evenly emarginate, with complete, marginal stria; mesometaventral stria absent; disk with only fine ground punctation. ***Metaventrite***: Metaventral disk impunctate at middle, with few fine punctures in front of metacoxae, lateral metaventral stria present, extending toward outer corner of metacoxa, slightly obsolete apically; postmesocoxal stria very short. ***Abdomen***: Abdominal ventrites finely punctate at middle, slightly more coarsely at sides; propygidium strongly transverse, about four times as wide as long, coarsely punctate, with punctures separated by slightly less than their diameters, a little denser toward sides; pygidium similarly coarsely punctate at base, punctures becoming finer, but also denser toward apex. ***Male***: 8^th^ tergite with deep basal emargination, ventrobasal processes nearly meeting, dorsally with fine, narrow median emargination; halves of 8^th^ sternite slightly separated along midline, apicomedial processes broadly lobate; 9^th^ tergite with acute, slightly recurved apices, ventrally with uniquely denticulate basal apodeme; spiculum gastrale (S9) broad in basal third, abruptly narrowed, broadening gradually toward head, apex broadly emarginate; 10^th^ tergite entire but weakly desclerotized along much of midline; aedeagus flattened throughout, sides weakly rounded, widest just apicad midpoint, apices meeting; median lobe slightly more than one-half tegmen length.

**Figure 7.**
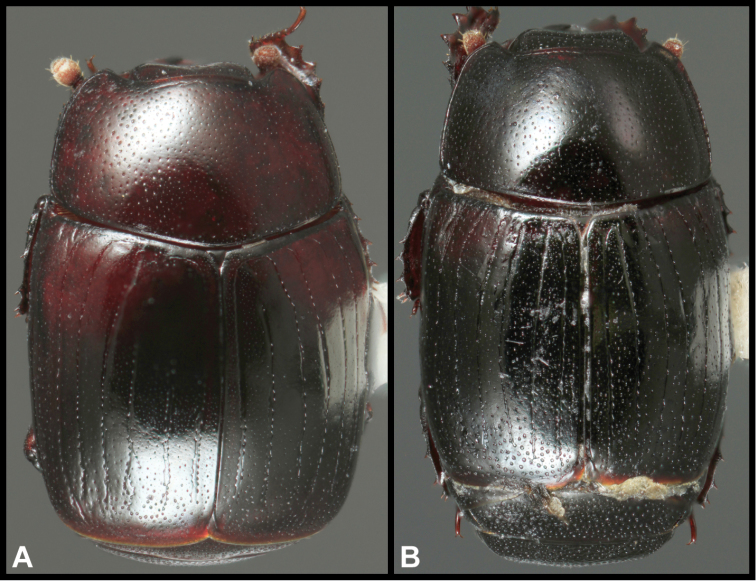
Dorsal habitus. **A** *Megalocraerus madrededios* **B** *Megalocraerus tiputini*.

#### Remarks.

This species is named for the rich region of Peru in which it is found. This species was included in the phylogenetic analysis of Caterino and Tishechkin (2015) as ‘*Megalocraerus*1’.

**Figure 8.**
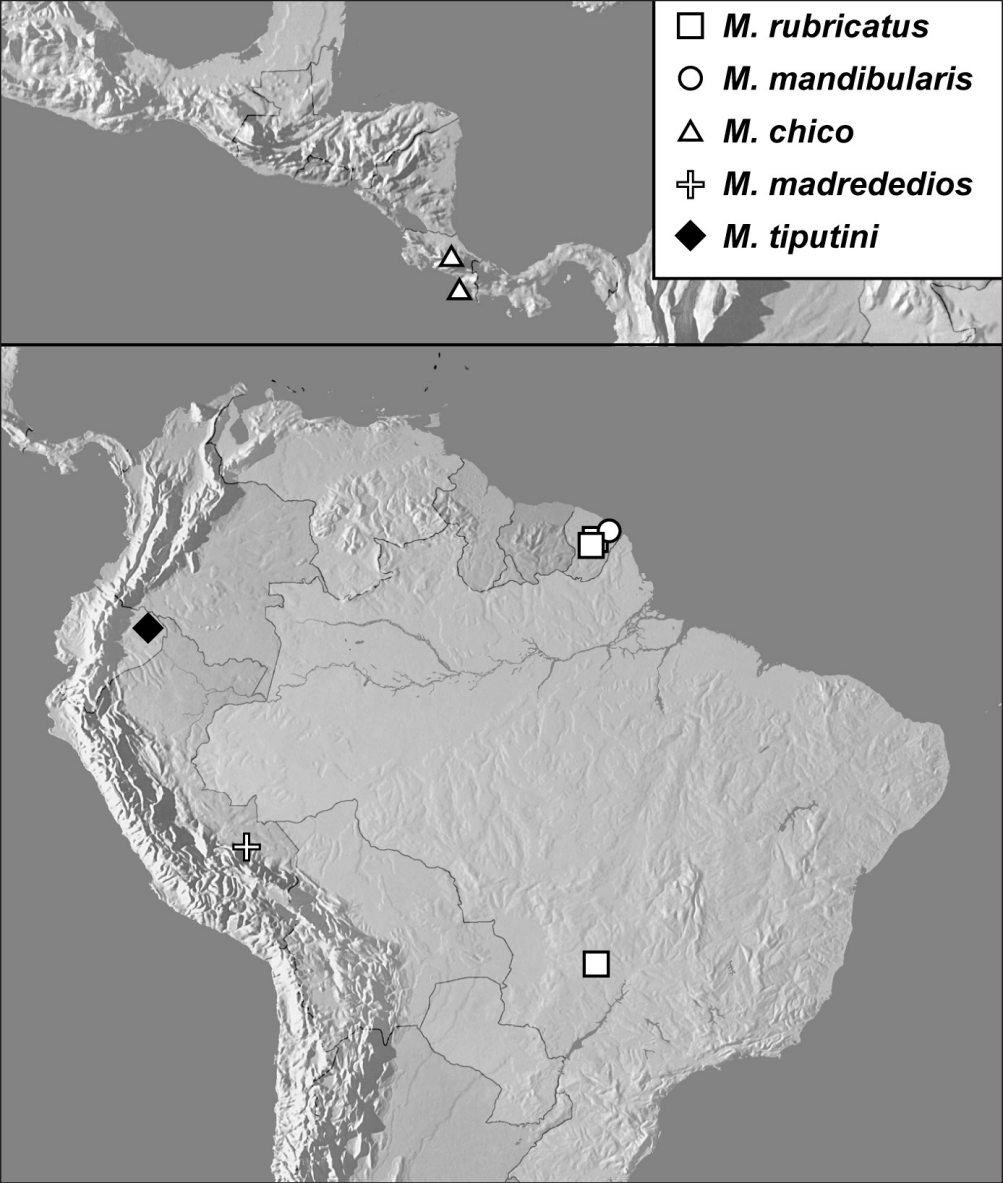
Map showing all collecting records for *Megalocraerus* spp.

### Megalocraerus tiputini sp. n.

Taxon classificationAnimaliaColeopteraHisteridae

http://zoobank.org/F0EF342E-532B-45AF-A602-E0CF3664511C

Figs 7B , 8

#### Type material.

**Holotype female: ECUADOR**: Orellana: Est. Biodiv. Tiputini, 0.6376°S, 76.1499°W, 2–9.vi.2011, FIT, M. Caterino & A. Tishechkin, DNA Extract MSC-2211, EXO-00738 (FMNH).

#### Diagnosis.

This species’ most distinctive character is its conspicuous metaventral punctation. It is also distinctly more densely punctate on the pygidia than other species. It is also unique (in the sole known specimen, at least) in having a complete 5^th^ dorsal stria joined to the sutural stria.

#### Description.

***Size***: Length 3.2 mm; width 2.3 mm; ***Body***: subparallel-sided, elongate, weakly subcylindrical; piceous. ***Head***: Frons finely and doubly punctate, with medium punctures separated by slightly more than their diameters against fine ground punctation, decreasing in size but slightly increasing in density anteromediad; frontal stria present along inner margins of eyes, bent inward above antennal insertions, broadly interrupted across epistoma; supraorbital stria absent; epistoma depressed, narrowing anterad; labrum minutely punctate; mandibles each with inner marginal tooth. ***Pronotum***: Pronotal sides weakly convergent from base to apical fourth, thence abruptly narrowing, marginal stria complete, continuous with distinct anterior marginal stria, lateral stria absent; pronotal disk finely and shallowly but densely punctate, slighlty sparser basomedially; basal margin with only few coarser punctures. ***Elytra***: Two complete epipleural stria present; subhumeral striae absent; dorsal striae 1–5 complete, the 5^th^ stria meeting basal arch of sutural stria; elytral disks with conspicuous ground punctation throughout, increasing in density apically. ***Prosternum***: Prosternal lobe evenly, somewhat narrow rounded, complete to sides, marginal stria obsolete at sides; prosternal keel with two complete carinal striae diverging slightly front and rear. ***Mesoventrite***: Anterior mesoventral margin evenly emarginate, with complete, marginal stria; mesometaventral stria absent; disk with fine secondary punctation. ***Metaventrite***: Metaventral disk finely but densely punctate at middle, more coarsely to sides, lateral metaventral stria present, curving laterad toward middle of metepisternum, slightly obsolete apically; postmesocoxal parallel but shorter. ***Abdomen***: Abdominal ventrites distinctly punctate throughout; propygidium transverse, about three times as wide as long, coarsely punctate, with punctures separated by about half their diameters, rather uniformly throughout; pygidium similarly coarsely punctate at base, diminished in size apically.

#### Remarks.

Although known from a sole female, this species is more than adequately distinct to describe. We name the species to honor the staff and facilities of the Tiputini Biodiversity Station, who have assisted us and countless other researchers in studying the biota of the region. This species was included in the phylogenetic analysis of Caterino and Tishechkin (2015) as ‘*Megalocraerus*4’.

### Unplaced specimens

**Brasil**: 1: [Rio de Janeiro] Corcovado-GB, 3.xii.1965, Moure-Seabra (UFPR).

**Remarks.** This specimen represents a distinct species based on male genitalia, with a broader and more dorsally ‘humped’ aedeagus than any other species (Fig. 6G, H) Unfortunately the body of this specimen was lost, aside from the male genitalia, so we refrain from describing it as new. We hope that additional collecting in the remaining Atlantic Forest around Rio de Janeiro will one day turn up additional material of this species. This species was included in the phylogenetic analysis of Caterino and Tishechkin (2015) as ‘*Megalocraerus*3’.

**French Guiana**: 1: Mont Tabulaire Itoupé, 3.022°N, 53.084°W, 400 m, 17.iii.2010, FIT, SEAG (CHND); 2: Nouragues, Inselberg Camp, 4.08°N, 52.68°W, 30.ix.2012, SEAG (CHND).

**Remarks.** These three specimens key to *Megalocraerus chico* described above, and share tibial characters with *Megalocraerus mandibularis*, but they are considerably larger than either, and may represent an undescribed species. However, all the specimens are poorly preserved, missing many parts, and are either female or lack genitalia (some genitalia were lost prior to mounting, and their sex is unknown). So we refrain from recognizing an additional species until better preserved material leads to a more conclusive assessment.

## Discussion

*Megalocraerus* has long represented an enigmatic lineage of rare beetles with unusual and confusing morphological attributes. One recent study (Caterino and Tishechkin 2015) has helped to clarify its phylogenetic position (although access to molecular-quality samples would be very useful), and the current study helps to better characterize its morphology and diversity. However, much remains unknown, particularly any hints as to its biology. Its rarity and unusual characters suggest something atypical for Exosternini, and we hope that microhabitat-focused collecting in areas of high diversity (esp. French Guiana, where 2 or 3 species are known to exist) will soon reveal more about these species’ life histories.

## Supplementary Material

XML Treatment for Megalocraerus

XML Treatment for Megalocraerus rubricatus

XML Treatment for Megalocraerus mandibularis

XML Treatment for Megalocraerus chico

XML Treatment for Megalocraerus madrededios

XML Treatment for Megalocraerus tiputini
